# Arteriovenous malformation Map2k1 mutation affects vasculogenesis

**DOI:** 10.1038/s41598-023-35301-6

**Published:** 2023-07-08

**Authors:** Christopher L. Sudduth, Patrick J. Smits, Matthew P. Vivero, Yu Sheng Cheng, Michal Ad, Dennis J. Konczyk, Joyce Bischoff, Matthew L. Warman, Arin K. Greene

**Affiliations:** 1 Department of Plastic and Oral Surgery, Boston Children’s Hospital, Harvard Medical School, 300 Longwood Ave., Boston, MA 02115 USA; 2 Department of Orthopedic Surgery, Boston Children’s Hospital, Harvard Medical School, Boston, MA USA; 3 Vascular Biology Program, Boston Children’s Hospital, Harvard Medical School, Boston, MA USA

**Keywords:** RNA, Translational research, Genetics research

## Abstract

Somatic activating *MAP2K1* mutations in endothelial cells (ECs) cause extracranial arteriovenous malformation (AVM). We previously reported the generation of a mouse line allowing inducible expression of constitutively active MAP2K1 (p.K57N) from the *Rosa* locus (*R26*^*GT-Map2k1-GFP*/+^) and showed, using *Tg-Cdh5CreER,* that EC expression of mutant MAP2K1 is sufficient for the development of vascular malformations in the brain, ear, and intestines. To gain further insight into the mechanism by which mutant MAP2K1 drives AVM development, we induced MAP2K1 (p.K57N) expression in ECs of postnatal-day-1 pups (P1) and investigated the changes in gene expression in P9 brain ECs by RNA-seq. We found that over-expression of MAP2K1 altered the transcript abundance of > 1600 genes. Several genes had > 20-fold changes between MAP2K1 expressing and wild-type ECs; the highest were *Col15a1* (39-fold) and *Itgb3* (24-fold). Increased expression of COL15A1 in *R26*^*GT-Map2k1-GFP*/+^; *Tg-Cdh5CreER*^+*/−*^ brain ECs was validated by immunostaining. Ontology showed that differentially expressed genes were involved in processes important for vasculogenesis (e.g., cell migration, adhesion, extracellular matrix organization, tube formation, angiogenesis). Understanding how these genes and pathways contribute to AVM formation will help identify targets for therapeutic intervention.

## Introduction

Arteriovenous malformation (AVM) is defined by a nidus of abnormal vessels forming direct connections between arteries and veins instead of a normal capillary network. Extracranial AVMs enlarge over time through four stages: Stage 1 (quiescence), Stage 2 (enlargement), Stage 3 (ulceration), Stage 4 (congestive heart failure)^1^. AVM morbidity includes disfigurement, pain, ulceration, bleeding, destruction of tissues, and congestive heart failure. Management consists of embolization of the affected vasculature and/or surgical resection. AVMs recur following these interventions and patients rarely are cured.

Extracranial AVM is most commonly caused by a somatic *MAP2K1* gain-of-function mutation in the endothelial cell (EC)^2^. MAP2K1 is a component of the RAS/MAP2K1 signaling pathway which is involved in numerous cellular and developmental processes^3^. MAP2K1 activates ERK1/2 which translocates to the nucleus where it stimulates expression of the transcription factors c-FOS, c-JUN, and c-MYC disrupting cell function^4^. Somatic activating mutations in upstream components of the MAP2K1 signaling pathway (KRAS, HRAS, BRAF) also can cause AVMs^5–8^. The RAS/MAP2K1 pathway is one of the most commonly mutated oncogenic pathways in cancer and the activating mutations present in AVMs are also often found within malignancies. Cancer related studies of the RAS/MAP2K1 pathway have led to the development of several FDA approved MEK1 inhibitors which could be repurposed as potential AVM treatments^9^. Recently, patients with severe extracranial AVMs have been managed off-label with the MEK inhibitor trametinib that was originally developed to treat melanoma^10–12^.

The purpose of this study was to perform RNA profiling to gain insight into how the endothelial MAP2K1 mutation leads to AVM formation and enlargement. We recently reported the generation of a mouse line that allows for inducible tissue specific expression of MAP2K1 (p.K57N) from the *Rosa* locus (*R26*^*GT-Map2k1-GFP*/+^)^13^. Postnatal topical tamoxifen activation of MAP2K1 expression resulted in the development of vascular malformations in the skin of the ear, intestines, and brain. These lesions share features with human Stage 1 extracranial AVMs: tortuous perfused vascular networks, enlarged blood vessels, presence of pericytes, mutant and wild-type ECs, increased p-ERK, and hemorrhage^13^. Our goal is to identify downstream pathways affected by the AVM endothelial MAP2K1 mutation in order to develop drugs for patients.

## Results

**Figure 1:**
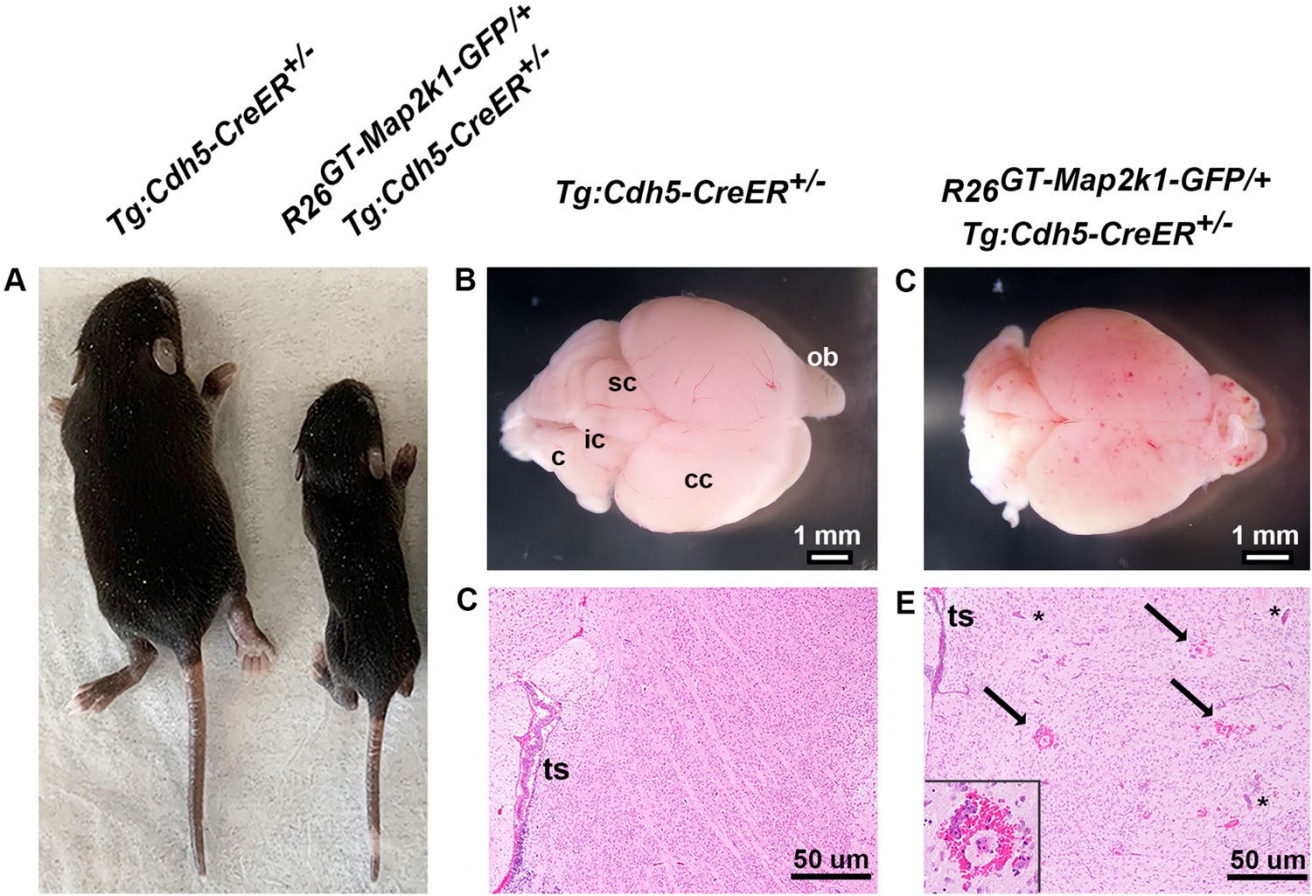
Expression of MAP2K1 (p.K57N) causes brain vascular malformations in a *R26*^*GT-Map2k1-GFP*/+^; *Tg:Cdh5-CreER*^+*/−*^ mouse line. (**A**) Pups at P9 after subcutaneous tamoxifen injection at P1. Notice the growth retardation in the *R26*^*GT-Map2k1-GFP*/+^; *Tg:Cdh5-CreER*^+*/−*^ pup. (**B**,**C**) Brains from the pups shown in (**A**). *R26*^*GT-Map2k1-GFP*/+^; *Tg:Cdh5-CreER*^+*/−*^ brains contain multifocal vascular malformations. (c) cerebellum, (ic) inferior colliculus, (sc) superior colliculus, (cc) cerebral cortex, and (ob) olfactory bulb. (**C**,**D**) Hematoxylin and eosin stained sections through the cerebral cortex demonstrate enlarged vessels (asterisks) and hemorrhage (arrows and insert) in the *R26*^*GT-Map2k1-GFP*/+^; *Tg:Cdh5-CreER*^+*/−*^ brain. Transverse sinus (ts).

P1 activation of MAP2K1 (p.K57N) expression in ECs by subcutaneous injection of tamoxifen caused lethargy and subsequent death of *R26*^*GT-Map2k1-GFP*/+^;*Tg-Cdh5CreER*^+*/−*^ pups between P10-12. To prevent detection of gene expression changes resulting from poor animal health, we analyzed the RNA profile of mutant MAP2K1 expressing and wild-type brain ECs at P9 before the earliest onset of lethargic behavior. Tamoxifen treated P9 *R26*^*GT-Map2k1-GFP*/+^;*Tg-Cdh5CreER*^+*/−*^ mice were growth retarded and their brains contained multifocal vascular malformations that were not present in control littermates (Fig. 1A–C). Histopathology illustrated numerous enlarged blood vessels and hemorrhage in the brain parenchyma in *R26*^*GT-Map2k1-GFP*/+^; *Tg-Cdh5CreER*^+*/−*^ mice, similar to the phenotype observed when mutant MAP2K1 expression is activated with topical tamoxifen (Fig. 1D,E)^13^.

Droplet digital PCR (ddPCR) showed that a recombined *R26*^*GT-Map2k1-GFP*/+^ allele was present in 20–28% of brain ECs extracted from tamoxifen treated P9 *R26*^*GT-Map2k1-GFP*/+^; *Tg-Cdh5CreER*^+*/−*^ mice (Supplementary Fig. 1). For our transcriptome analysis we used RNA extracted from brain ECs from four wild type and four *R26*^*GT-Map2k1-GFP*/+^; *Tg-Cdh5CreER*^+*/−*^ P9 pups. Integrity numbers for each sample were high (8.9–9.8). RNA seq yielded an average of 36 million reads (± 4.2 million) per sample, with > 91% of reads mapping to the reference genome. To assess the purity of the extracted ECs we analyzed the presence of non-EC specific transcripts in the dataset. Reads for *Gfap* (astrocytes; ENSMUSG00000020932), *Higd1b* (pericytes; ENSMUSG00000020928), *Cnn1* (smooth muscle myocytes; ENSMUSG00000001349), and *Dcn* (fibroblasts; ENSMUSG00000019929) were low (Supplementary Fig. 2).

**Figure 2:**
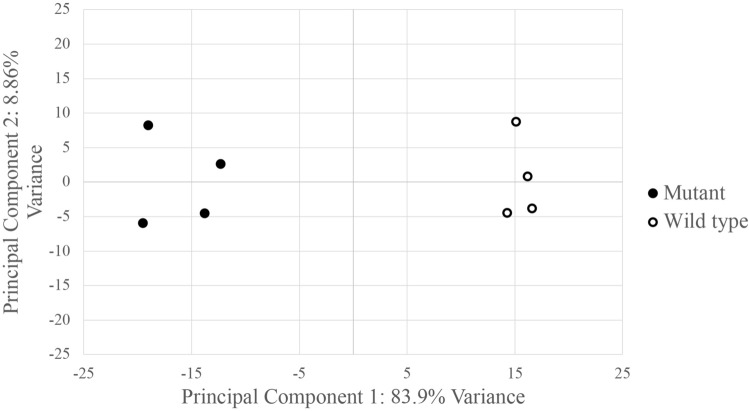
Principal component analysis shows *R26*^*GT-Map2k1-GFP*/+^; *Tg:Cdh5-CreER*^+*/−*^ ECs have a distinct molecular signature.

**Figure 3:**
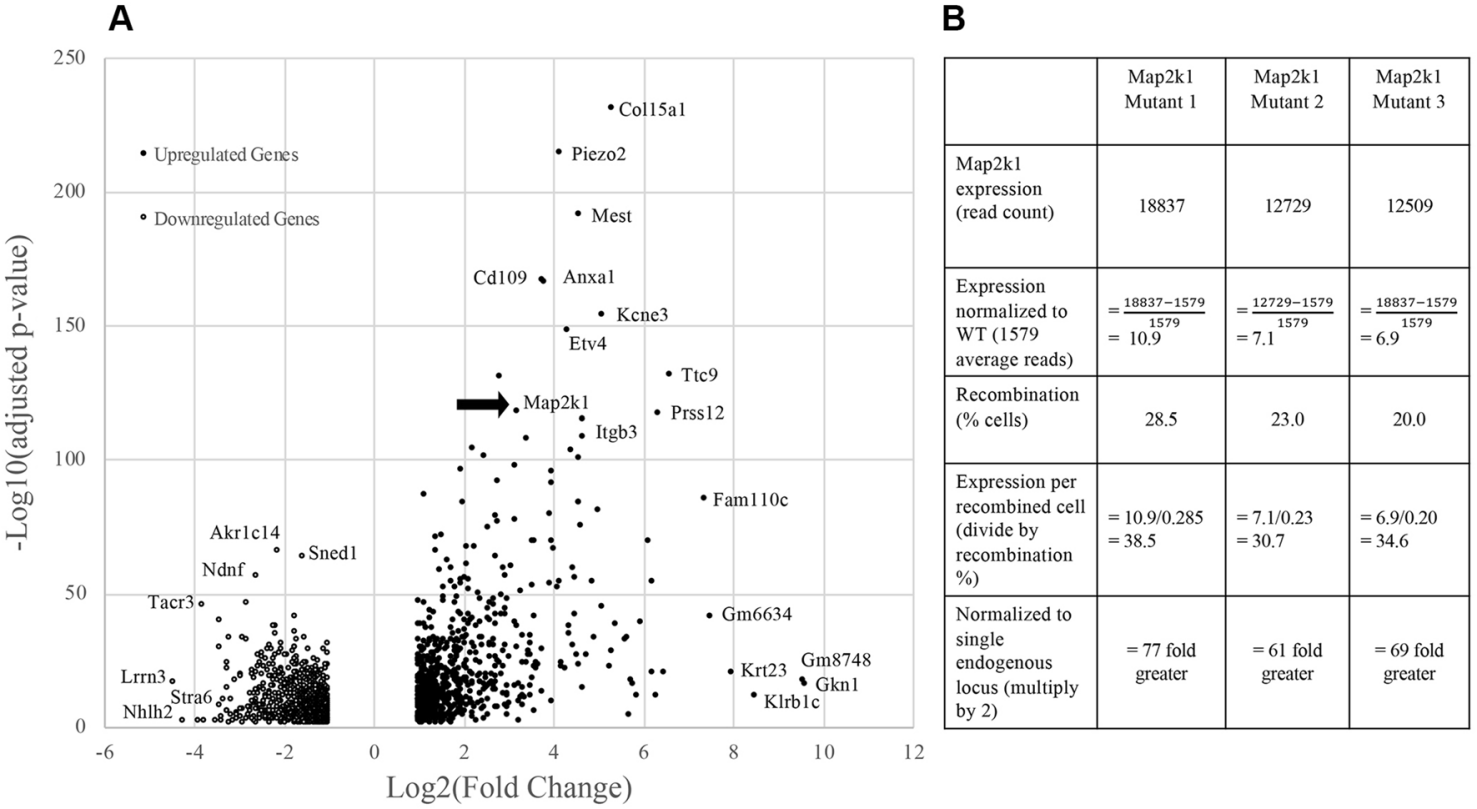
(**A**) Volcano plot for the 1,677 significantly differentially expressed genes. Adjusted p-value (y-axis) is plotted against Log(2) fold change (x-axis). The most significantly affected gene is *Col15a1.* Note that *Map2k1* transcripts are increased (arrow). (**B**) Comparison of *Map2k1 (p.K57N)* expression levels to wild type *Map2k1* in brain ECs with a recombined *R26*^*GT-Map2k1-GFP*/+^ allele. Expression from the activated *R26*^*GT-Map2k1-GFP*^ allele is on average 69 × greater than expression from an endogenous *Map2k1* allele.

Principal component analysis grouped the *R26*^*GT-Map2k1-GFP*/+^; *Tg-Cdh5CreER*^+*/−*^ RNA seq datasets separately from the wild-type datasets (Fig. 2). Because EC cell recovery, RNA extraction, library preparation, and sequencing were performed on all samples at the same time, the difference between mutant and wild-type EC datasets likely represents biologic differences rather than artifactual batch effects. Differential gene expression analysis identified 790 genes whose transcript abundance increased, and 887 genes whose transcript abundance decreased (Fig. 3A and Supplementary Tables 1, 2). *Map2k1* was identified among the increased transcripts. To estimate the relative expression of the recombined *R26*^*GT-Map2k1-GFP*/+^ allele compared to the two endogenous alleles, we counted mutant versus wild type reads and normalized for the % of recombined cells. This analysis determined that mRNA expression from a recombined *R26*^*GT-Map2k1-GFP*/+^allele was on average 69-fold higher than endogenous *Map2k1* expression (Fig. 3B).

**Figure 4:**
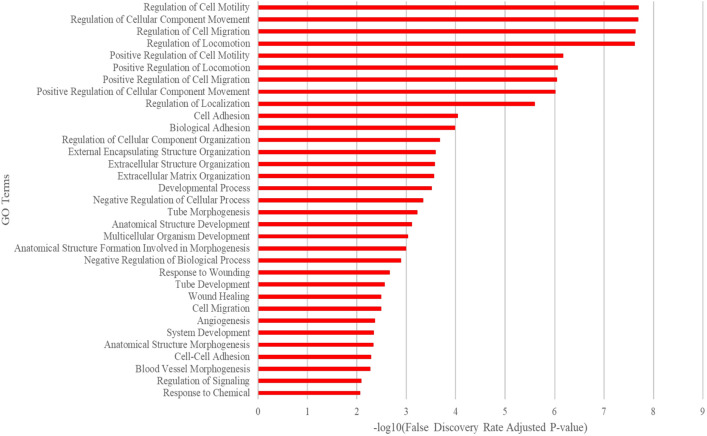
Ontology using the top 200 differentially expressed genes ranked by adjusted p-value. The most impacted pathways were cell migration, cell adhesion, and matrix organization associated with tube morphogenesis, angiogenesis, and blood vessel development.

**Figure 5:**
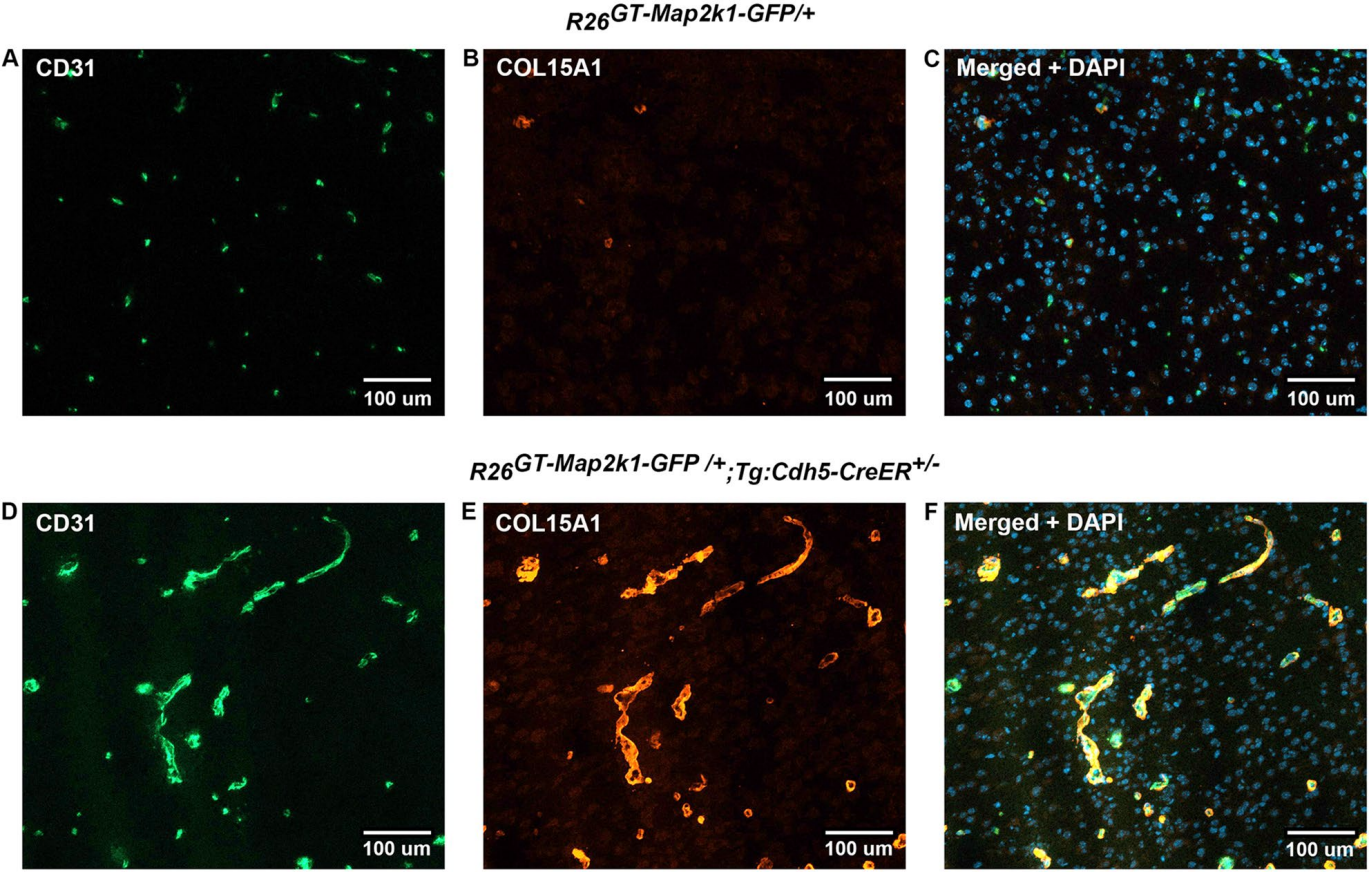
Endothelial cell *Map2k1* mutation causes increased COL15A1 protein production. Top panels (**A**–**C**) Immunostaining of P9 *R26*^*GT-Map2k1-GFP*/+^ brain sections. Bottom panels (**D–F**) Immunostaining of P9 *R26*^*GT-Map2k1-GFP*/+^; *Tg:Cdh5-CreER*^+*/−*^ brain sections. (**A**,**D**) CD-31; (**B**,**E**) COL15A1; (**C**,**F**) CD-31 + COL15A1 + DAPI. Note abnormal vasculature and COL15A1 expression in the *R26*^*GT-Map2k1-GFP*/+^; *Tg:Cdh5-CreER*^+*/−*^ brain.

Gene Ontology Resource was used to identify pathways affected in ECs by mutant MAP2K1. The top 200 differentially expressed genes showed significant alterations in cell migration, adhesion, matrix organization, tube morphogenesis, angiogenesis, and blood vessel development (Fig. 4). Notable among the genes with increased expression were the basement membrane collagen *Col15a1* (39-fold increase, padj ~ 10^–236^), the cell adhesion molecule *Itgb3* (24-fold increase, padj ~ 10^–109^), the voltage gated stretch receptor *Piezo2* (17-fold increase, padj ~ 10^–215^), and the GPI-anchored cell surface protein *Cd109* (13-fold increase, padj ~ 10^–168^). Fifteen other genes with low adjusted p values had > tenfold expression changes, and 85 had > four-fold changes (Supplementary Table 1). One gene, *Stra6,* had its transcript abundance decrease > tenfold (padj ~ 10^–9^) and 52 other genes had their abundances decrease > four-fold (Supplementary Table 2). To validate the RNA sequencing findings, we immunostained brain sections of control and *R26*^*GT-Map2k1-GFP*/+^; *Tg-Cdh5CreER*^+*/−*^ animals for COL15A1, the highest upregulated gene. *R26*^*GT-Map2k1-GFP*/+^; *Tg-Cdh5CreER*^+*/−*^ brains showed a strong increase in COL15A1 positive ECs compared to non-mutant brains (Fig. 5).

## Discussion

Previous AVM RNA sequencing studies have involved either human brain AVM specimens which do not contain *MAP2K1* mutations or in vitro engineered KRAS (p.G12D) overexpressing ECs^14–17^. In these systems genes involved in inflammation, cell migration, extracellular matrix remodeling, and angiogenesis are differentially expressed. Our study illustrates the transcriptional profiling of ECs from MAP2K1 (p.K57N) induced brain vascular malformations using a *R26*^*GT-Map2k1-GFP*/+^; *Tg-Cdh5CreER*^+*/−*^ mouse line. Our RNA sequencing results are consistent with single cell RNA data from human brain AVMs which also exhibited increased *COL15A1*, *PGF*, *PLVAP*, *STC1*, *ANGPT2* and decreased *MFSD2A*, *SLC16A1*, *SLC38A5*^16,17^. Advantages of our model are that it is in-vivo, has a known mutation, and produces an obvious phenotype. Disadvantages of our mouse line include the possibility that gene expression changes might be different if mutant MAP2K1 was expressed at endogenous levels or if changes were compared to mice expressing wild type MAP2K1 protein from the *Rosa* locus.

The transcriptome changes in MAP2K1 expressing ECs provides insights into the mechanisms that contribute to AVM formation. Overexpression of MAP2K1 resulted in more than 1600 genes whose expression was significantly different between mutant and non-mutant ECs. None of the genes alone appear to be necessary and sufficient to produce an AVM. However, many of the overexpressed transcripts in our dataset promote vasculogenesis. For example, upregulated *Esm1* and *Vav3* expression may cause dysregulated EC migration and filopodia extension. ECs in *Esm1* null mice exhibit delayed vascular outgrowth and reduced filopodia extension^18^. Microvascular ECs overexpressing VAV3 demonstrate increased migration and assembly while embryonic fibroblasts deficient in VAV3 have reduced filopodia extension^19^. COL15A1 forms a proangiogenic scaffold for ECs. ECs cultured with fibroblasts overexpressing COL15A1 have increased migration and tube formation^20^. ITGB3 is a heterodimeric adhesion receptor that interacts with the extracellular matrix and is highly expressed in vessels undergoing pathological angiogenesis^21^. ROBO1, CDH13, PGF, ANGPT2, and FGFR1 increase EC proliferation, migration, and tube formation^22–25^. Dysregulated expression of multiple proangiogenic factors in MAP2K1 expressing ECs may result in their inability to form normal capillary beds which could lead to arteriovenous shunts.

It is not feasible to obtain mice with a global or conditional knockout allele for every differentially expressed gene to test that gene’s role in producing vascular malformations when mutant *Map2k1* is expressed. However, it is possible to use genes with both large fold-changes in transcript abundance and increased protein expression for cell-based assays of mutant *Map2k1* effects. For example, CD109, whose transcript is 13-fold more abundant in mutant ECs, can be used to flow sort ECs in perturb-seq experiments. As an alternative to antibody-mediated flow sorting, a fluorescent reporter could be knocked into the *Col15a1* locus, whose transcript is 39-fold more abundant in MAP2K1 mutant ECs.

Our results showing that the *Map2k1* mutation in ECs affects vasculogenesis genes and pathways contributes to our understanding of AVMs pathogenesis. The activating *MAP2K1* EC mutation may cause AVM enlargement through abnormal blood vessel production that secondarily causes overgrowth of tissues. This would explain the finding that human AVMs enlarge by a cell non-autonomous mechanism^26^. Drugs that inhibit EC migration, adhesion, and angiogenesis may prove beneficial to patients with AVMs. Anti-angiogenic medications might prevent enlargement of AVMs as well as reduce their recurrence following embolization and resection. Combining anti-angiogenic drugs with MAP2K1 inhibitors for AVM may prove synergistic, improving the efficacy of drugs like trametinib and reducing its toxicity.

## Methods

### Conditional expression of MAP2K1 (p.K57N) in mouse endothelial cells

Animal experiments were approved by the Boston Children’s Hospital’s Institutional Animal Care and Use Committee (Protocol 00001349). All methods reported are in accordance with the relevant guidelines and regulations including the ARRIVE guidelines^27^. *R26*^*GT-Map2k1-GFP*^ and *Tg-Cdh5CreER* mice were previously described^13,28^. Both strains were maintained by backcrossing with C57BL/6J animals (Jax: 000664). Male *Tg-Cdh5CreER* heterozygous animals were mated with *R26*^*GT-Map2k1-GFP*^ heterozygous females. To induce CRE mediated recombination in resulting litters we administered tamoxifen (100 µg: 20 µl of a five mg/ml tamoxifen solution in sesame oil) by subcutaneous injection at postnatal day one (P1). Pups were euthanized at P9 by decapitation and brain ECs were extracted. Tail DNA from each pup was genotyped for *Map2k1* (Ex7-FP: 5′-ggagctactgtttggatgccatgtg-3′ and Ex11-RP: 5′-ctgggctggttaagcccaatggtg-3′; amplicon size 342 bp) and *CreER* (FP: 5′-caataccggagatcatgcaagctg-3′ and RP: 5′-aggcacattctagaaggtggacctg-3′; amplicon size 429 bp). PCR cycle: 94 °C 3′ =  > 36× (94 °C 15″ =  > 61 °C 15″ =  > 72 °C 30″) =  > 72 °C 7′.

### Study design

All pups appeared healthy at P9, and none were excluded. Wild type pups were randomized within litters and selected to match the number of mutant pups from the same litter (e.g., two mutants and two random wild type pups from litter one). Because of the phenotypic changes in *R26*^*GT-Map2k1-GFP*/+^; *Tg-Cdh5CreER*^+*/−*^ pups, blinding and masking was not possible. The main outcome measure was differences in gene expression between wild type and *R26*^*GT-Map2k1-GFP*/+^; *Tg-Cdh5CreER*^+*/−*^ mice assessed by RNA sequencing.

### Histology and immunostaining

Mouse brains (n = four for control wild type, *R26*^*GT-Map2k1-GFP*/+^ or *Tg-Cdh5CreER*^+*/−*^ and n = 4 for mutant *R26*^*GT-Map2k1-GFP*/+^; *Tg-Cdh5CreER*^+*/−*^) were fixed in formalin, embedded in paraffin, sectioned (seven µm), and hematoxylin/eosin stained. Tissue undergoing immunostaining were flash frozen in OCT and stored at − 80 °C. Seven µm cryo-sections were cut. Primary antibodies used: rabbit anti-mouse COL15A1 (Gift from Dr. Karppinen, University of Oulu, Finland) (1/500) and rat anti-mouse CD31 (1/500) (BD Pharmigen: 553370). Secondary antibodies used: donkey anti-mouse IgG (H + L) Alexa Fluor 488 (Abcam 150105, 1/200); donkey anti-rabbit IgG (H + L) rhodamine red-X (1/500) (Jackson ImmunoResearch 712-295-150). Sections were mounted with DAPI Fluoromount-G (SouthernBiotech). Images of sections were obtained using an Eclipse 80i microscope (Nikon) with a Spot model 25.4 camera and Spot 5.2 software (Diagnostic Instruments). Confocal images were captured using a Zeiss LSM 800 confocal microscope and Zen Blue software version 2.5.

### Endothelial cell isolation

ECs were isolated as previously described^6^. Briefly, whole brain was minced with a scalpel, digested with collagenase A (Roche # 11088793001) at 37 °C for 1.5 h, and passed through an 18-gauge needle. Cells were filtered through a 70-μm strainer and incubated for 15 min at 4 °C with anti-CD31 antibody (BD Biosciences #553370) that had been previously conjugated to magnetic dynabeads (ThermoFisher #11035) following the manufacturer’s protocol. Non-bound cells were removed by washing three times with 0.1% BSA in PBS for one minute/wash at room temperature. The bound cells were resuspended in PBS and 1/20th of the suspension was kept for DNA extraction.

### Droplet digital PCR (ddPCR)

The *R26*^*GT-Map2k1-GFP*^ allele contains from 5′ to 3′: a CAG promoter, a LoxP flanked stop cassette (gene trap), the *Map2k1* (p.K57N) cDNA, an IRES-GFP cassette and a poly A signal^13^. DNA was extracted from isolated brain ECs using the DNeasy blood and tissue kit (Qiagen #69506). To detect the non-recombined *R26*^*GT-Map2k1-GFP*^ allele, a ddPCR assay was designed using a forward primer (FP) immediately upstream of the 5′ LoxP side of gene trap (5′-cgtgctggttattgtgctgtc-3′) and a reverse primer (RP) inside the gene trap (5′-gcgttggctacccgtgata-3′). The resulting amplicon size was 278 bp. A gene trap specific probe (5hex/caacacccgtgcgttttattctgt/3IABkFQ) was contained within the amplicon. To detect the recombined *R26*^*GT-Map2k1-GFP*^ allele a ddPCR assay was designed using the same FP in combination with a RP located within the *Map2k1* (p.K57N) cDNA (5′-gatctccaggtggatcagctt-3′). The resulting amplicon size was 2912 bp when the gene trap was present and 406 bp when the gene trap was removed by recombination. The amplicon contained the *Map2k1* cDNA specific probe 56-FAM/gagtggtcttcaaggtctcccaca/3IABkFQ. Primers and probes were obtained from Integrated DNA Technologies. PCR cycle used: 95 °C 10′ =  > 40× (94 °C 30″ =  > 60 °C 60″ =  > 72 °C 30″) =  > 98 °C 10′ (ramp time: 1.2 °C/s). The extension step of 30″ prevented amplification of the 2912 bp non-recombined fragment, making the *Map2k1* probe specific for the recombined allele. Reactions were performed in triplicate.

### RNA sequencing

RNA sequencing was performed on RNA extraction and library construction by GeneWiz (South Plainfield, NJ, USA). RNA quantity was determined using Qubit and assessed with the Agilent RNA 60,000 Nano kit. RNA libraries were made with poly(A^+^) RNA and sequenced on an Illumina HiSeq with 2 × 150 bp reads at ~ 40 million reads per sample. Analysis was performed by GeneWiz. Sequence reads were trimmed to remove adapter sequences and nucleotides with poor quality using Trimmomatic v.0.36. The trimmed reads were mapped to the Mus musculus GRCm38 reference genome available on ENSEMBL using the STAR aligner v.2.5.2b. Reads were normalized using DESeq2’s median of ratios, a sample-specific normalization method determined by the median ratio of gene counts relative to geometric mean per gene. Principal component analysis was performed to assess similarities and differences across the samples.

Differential gene expression analysis was carried out using DESeq2. We focused our attention on the top 80% of these ranked by average normalized read counts across all eight samples. RNA sequencing data are available in the NCBI gene expression omnibus (GEO) data repository.

*Map2k1* expression levels from a recombined *R26*^*GT-Map2k1-GFP*^ allele was calculated for three *R26*^*GT-Map2k1-GFP*/+^; *Tg-Cdh5CreER*^+*/−*^ mice. *Map2k1* transcript read numbers from *R26*^*GT-Map2k1-GFP*/+^; *Tg-Cdh5CreER*^+*/−*^ brain ECs were normalized for average *Map2k1* transcript read number in wild type brain ECs (1579) and adjusted for their respective recombination efficiency. Read numbers were then normalized to expression from one endogenous *Map2k1* allele.

### Statistical methods

Significantly differentially expressed genes had adjusted p values < 0.05 (after controlling for multiple comparisons using the Benjamani–Hochberg Method, and mean log2-fold change > or < 1). Pathway analysis using the top 200 differentially expressed genes (ranked by adjusted p value) was performed using the Gene Ontology Resource^29^.

## Supplementary Information

Supplementary Figure 1.

Supplementary Figure 2.

Supplementary Table 1.

Supplementary Table 2.

## Data Availability

The datasets generated and/or analyzed during the current study has been submitted to the Gene Expression Omnibus repository, accession number: GSE226484 (https://www.ncbi.nlm.nih.gov/geo/query/acc.cgi?acc=GSE226484).
