# Supplementary figures and images for: Arteriovenous malformation Map2k1 mutation affects vasculogenesis
Source: Sci Rep. 2023 Jul 8;13:11074. doi: 10.1038/s41598-023-35301-6 (PMC10329712; doi:10.1038/s41598-023-35301-6)

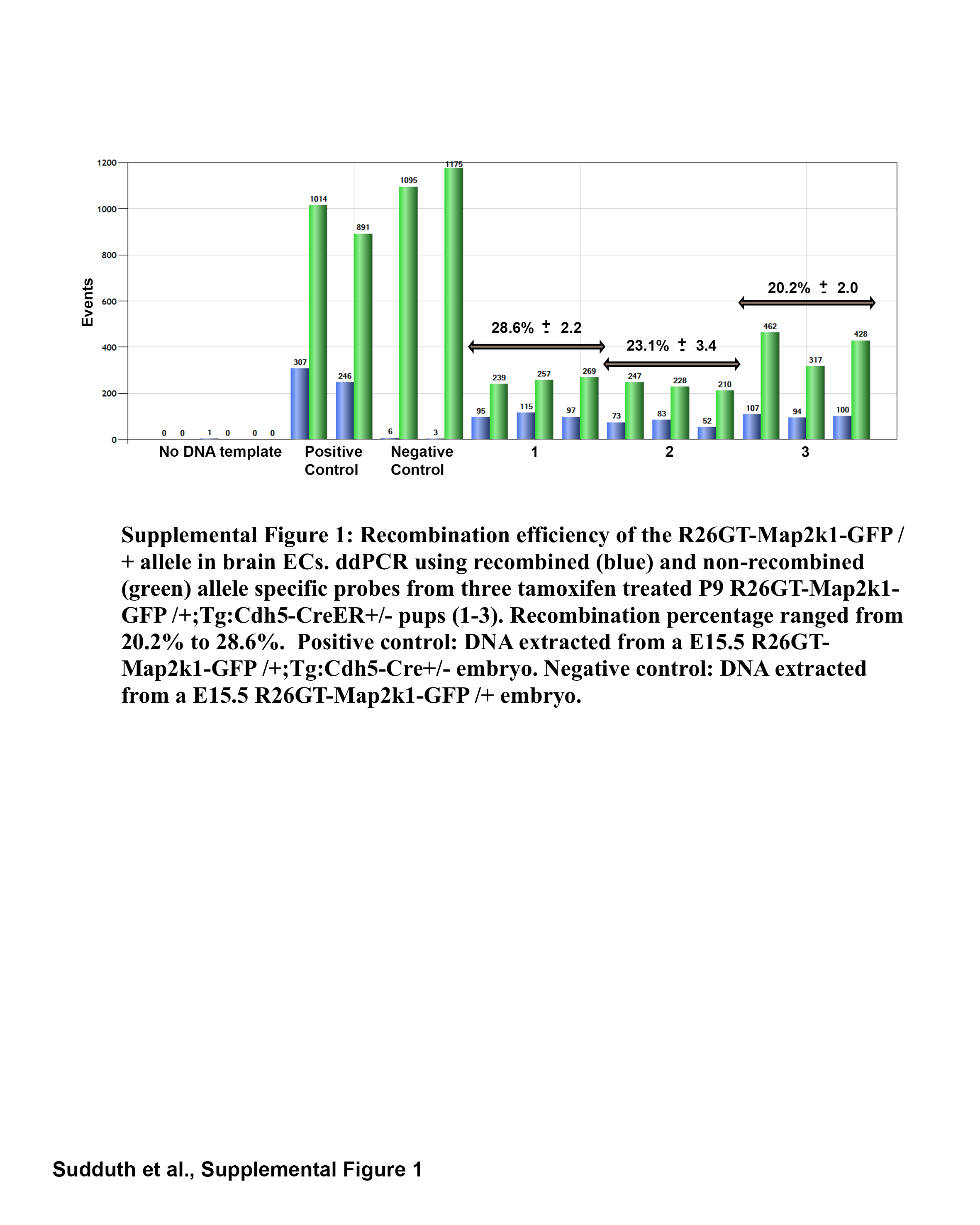

Supplement: Supplementary file 1 — Supplementary Figure 1. [file 41598_2023_35301_MOESM1_ESM.jpg]

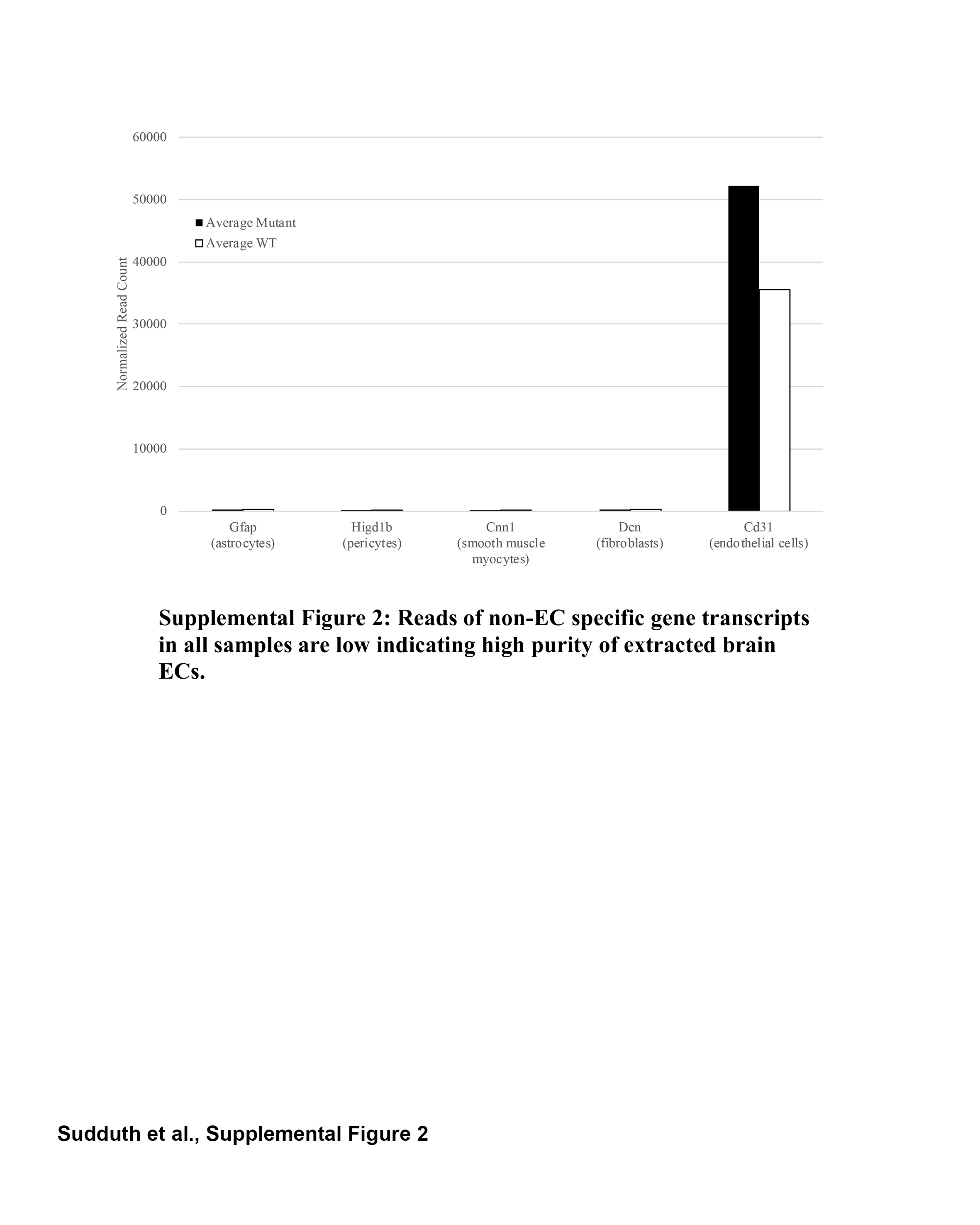

Supplement: Supplementary file 2 — Supplementary Figure 2. [file 41598_2023_35301_MOESM2_ESM.jpg]
